# Recovery of off-state stress-induced damage in FET-type gas sensor using self-curing method

**DOI:** 10.1186/s11671-023-03801-z

**Published:** 2023-02-25

**Authors:** Wonjun Shin, Yujeong Jeong, Mingyu Kim, Jungsoo Lee, Ryun-Han Koo, Seongbin Hong, Gyuweon Jung, Jae-Joon Kim, Jong-Ho Lee

**Affiliations:** Department of Electrical and Computer Engineering, Inter-University Semiconductor Research Center, Seoul National University, Seoul, 08826 South Korea

**Keywords:** In_2_O_3_, Horizontal floating-gate FET-type gas sensor, 1/*f* noise, Low-frequency noise, Self-curing

## Abstract

The need for high-performance gas sensors is driven by concerns over indoor and outdoor air quality, and industrial gas leaks. Due to their structural diversity, vast surface area, and geometric tunability, metal oxides show significant potential for the development of gas sensing systems. Despite the fact that several previous reports have successfully acquired a suitable response to various types of target gases, it remains difficult to maintain the reliability of metal oxide-based gas sensors. In particular, the degradation of the sensor platform under repetitive operation, such as off-state stress (OSS) causes significant reliability issues. We investigate the impact of OSS on the gas sensing performances, including response, low-frequency noise, and signal-to-noise ratio of horizontal floating-gate field-effect-transistor (FET)-type gas sensors. The 1/*f* noise is increased after the OSS is applied to the sensor because the gate oxide is damaged by hot holes. Therefore, the SNR of the sensor is degraded by the OSS. We applied a self-curing method based on a PN-junction forward current at the body–drain junction to repair the damaged gate oxide and improve the reliability of the sensor. It has been demonstrated that the SNR degradation caused by the OSS can be successfully recovered by the self-curing method.

## Introduction

Recent years have witnessed a significant rise in demand for high-performance gas sensors. From conventional uses such as indoor/outdoor air quality monitoring, applications of gas sensors have expanded to include medical diagnosis and food quality control [1–5]. Among different types of gas sensors, metal oxide (MOX)-based gas sensors have merits in terms of the simple fabrication process, low cost, simplicity in use, and a large number of detectable gases [6, 7]. Despite the numerous advantages, MOX-based gas sensors still suffer from poor stability and reliability due to their susceptibility to temperature and humidity [8].

Accordingly, there are previous studies aimed at enhancing stability and long-term reliability. Most of the previous studies focused on improving the reliability of MOX sensing materials. Han et al. demonstrated that the optimization of the metal-to-oxygen ratio in the SnO_2_ could achieve good stability at a temperature above 350 °C [9]. Postica et al. reported that coating ZnO with an ultra-thin layer of SiO_2_ improves long-term stability and minimizes the humidity effects [10]. Besides these studies, various parameters are known to affect the stability and long-term reliability of MOX sensing materials. For example, small particles degrade long-term stability. This problem may be partly resolved by preparing the nanoparticles in a slurry form and then depositing them directly onto the microelectrodes by ink-jet printing and micro-dispensing. In addition, it is difficult to achieve thermal stability of the particle with a small size. This issue can be overcome to some degree by using sensors that are functional at low temperatures [11].

**Fig. 1:**
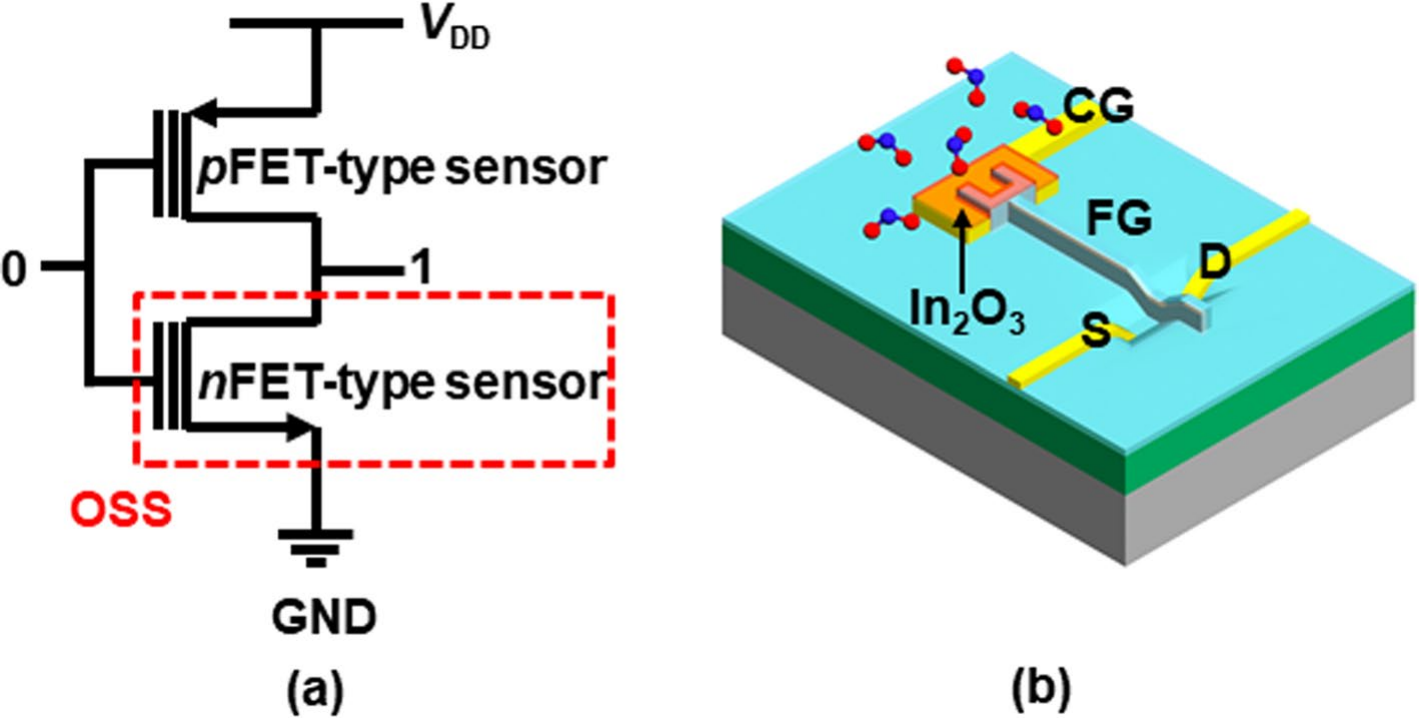
**a** Schematic of sensitive amplifier composed of *n*FET- and *p*FET-type gas sensors. The *n*FET is exposed to the OSS when the input signal is ‘0’. **b** Schematic of the 3D bird’s eye view of the *n*FET-type gas sensor

However, the reliability of the sensor is influenced not only by the sensing material (MOX), but also by the transducer of the sensor [12–14]. In particular, when configuring a sensitive amplifier circuit with *n*- and *p*-channel FET-type (*n*FET and *p*FET) gas sensors, the *n*FET-type gas sensor is exposed to iterative off-state stress (OSS) during the circuit operation [15–17]. When the input signal of the sensing amplifier circuit is low, the OSS is applied to the *n*FET (*V*_G_ = GND, *V*_D_ = *V*_DD_, *V*_S_ = GND, *V*_B_ = GND), as shown in Fig. 1a. It has been reported that the OSS degrades the gate oxide of the FET by generating interface and bulk traps [15]. When the 1/*f* noise is increased by the damaged gate oxide, the signal-to-noise ratio (SNR) and limit of detection (LOD) of the FET-type gas sensor are degraded because the FET transducer determines the low-frequency noise (LFN) characteristics [18–20]. Therefore, the impact of OSS on the sensing performances of the *n*FET-type gas sensor should be investigated.

In addition, the method to improve the degradation induced by the OSS should be provided. Recently, self-curing methods that utilize the internal operation of the FETs, including PN-junction forward current and punch-through current, have been proposed to improve the reliability of the FETs [21–23]. Unlike global forming gas annealing, electrothermal annealing, also known as the self-curing method, can be effectively applied to a unit device without the need for bulky equipment. The self-curing methods make use of the Joule heat (JH) that is inherently generated during the operation of FETs. JH is used to cure the defects inside the gate oxide with the same principle as thermal annealing. Recent reports demonstrated the JH can be induced by PN-junction forward current, gate-induced drain current leakage, and punch-through current [21–23]. Self-curing methods have significant advantages over furnace annealing because they can repair the damage even after packaging without affecting the interconnection or device parameters of other devices. In this regard, the self-curing methods have been applied to various applications, including memory devices [24]. However, its application has not been demonstrated in sensor applications despite its advantage and potential.

Following the above discussion, in this work, we investigate the impact of OSS on the gas sensing, LFN, and SNR of the *n*FET-type gas sensor with horizontal floating gate (FG). The In_2_O_3_ is used as a sensing material, and NO_2_ is used as a target gas. It is revealed that the gate oxide is degraded by the band-to-band tunneling (BTBT) due to the increased vertical electric field at the gate-drain overlap. After the OSS is applied, the magnitude of the 1/*f* noise is significantly increased, degrading the SNR and LOD of the sensor. In order to cure the damaged gate oxide, the self-curing method based on PN-junction forward current is applied. The proposed self-curing method represents a major advancement in gas sensor research by presenting a novel method to improve the reliability of gas sensors.

## Results and discussion

In a sensitive amplifier with *n*FET and *p*FET-type gas sensors, the *n*FET-type gas sensor is exposed to the OSS when the input signal is low, as shown in Fig. 1a. Figure 1b shows the schematic of the 3D bird’s eye view of the *n*FET-type gas sensor. The width/length (*W*/*L*) of the FET channel is 1 μm/0.6 μm. The detailed fabrication process of the sensor is explained in [13]. The simplified explanation of the sensor fabrication process is as follows: The devices are isolated using the local oxidation of silicon technique in an *n*-type Si wafer. A 10 nm-thick SiO_2_ is grown using dry oxidation as a gate oxide. A 350 nm-thick *in-situ* doped *n*^+^ polycrystalline Si is formed as FG. Source and drain are formed using self-align. The SiO_2_/Si_3_N_4_/SiO_2_ is successively formed as a passivation layer. Using a lift-off process, successive layers of Ti (30 nm), TiN (20 nm), and Al (100 nm) are deposited and patterned to form the electrodes for the drain, source, and body. These metal layers are also used as a control gate (CG). Finally, a 15-nm-thick *n*-type semiconducting In_2_O_3_ film is deposited using a sputtering method. Note that a detailed fabrication process of the sensor is explained in Ref. 13. In this work, the OSS is applied at 125 °C for the acceleration test. The *V*_D_ is set to OSS stress bias (*V*_D,OSS_), and the other terminals are grounded for the OSS. The stress time (*t*_OSS_) and *V*_D,OSS_ are changed to quantitatively investigate the impact of OSS.

**Fig. 2:**
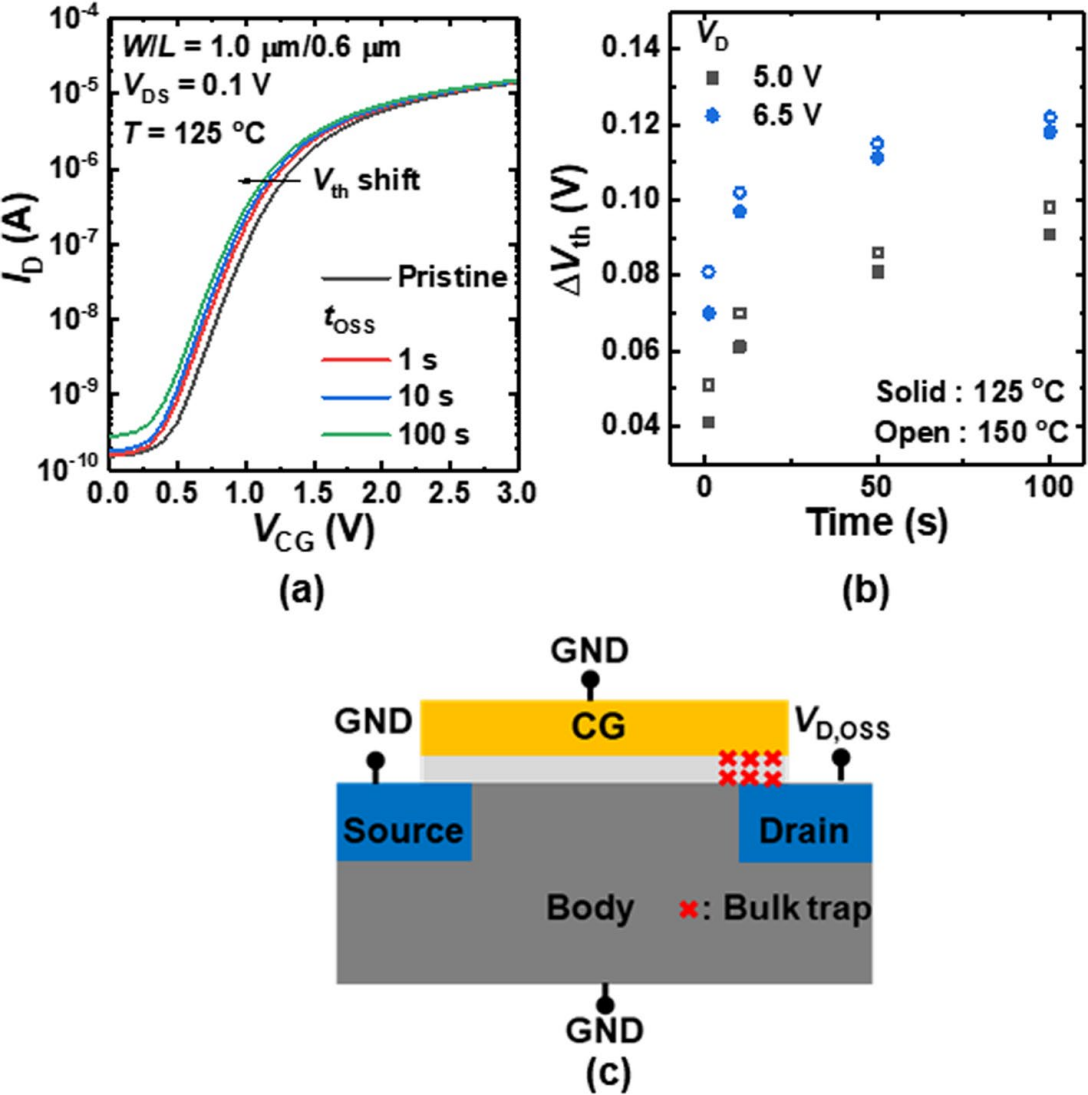
**a** *I*_D_–*V*_CG_ of the sensor as a parameter of *t*_OSS_ measured at 125 °C (Solid symbols) and 150 °C (Open symbols). **b** Δ*V*_th_ versus *t*_OSS_ at *V*_D,OSS_ of 5.0 V and 6.5 V. **c** Schematic illustration of the OSS-induced gate oxide degradation

Figure 2a shows the transfer characteristics (*I*_D_–*V*_CG_) of the *n*FET-type gas sensor as a parameter of *t*_OSS_. Note that the *V*_D,OSS_ is set at 6.5 V. The threshold voltage (*V*_th_) of the sensor is shifted to a negative direction with an increase in *t*_OSS_. As the electrical field is increased by the *V*_D,OSS_ at the gate/drain overlap region, the valence band electrons tunnel to the conduction band. The strong electric field causes these electrons to gain energy, inducing impact ionization to generate electron/hole pairs. The energetic holes move toward the gate along the electric field and these holes are eventually injected into the gate oxide, causing a negative *V*_th_ shift of the sensor. Figure 2b shows the *V*_th_ shift (Δ*V*_th_) versus *t*_OSS_ at *V*_D,OSS_ of 5.0 V and 6.5 V measured at 125 °C (solid symbol) and 150 °C (open symbol). Figure 2c schematically illustrates the mechanism of OSS-induced gate oxide degradation. During the OSS, hot holes cause traps and defects inside the gate oxide. It has been reported that the Si–O bonds are broken by the hot holes during the OSS [15]. When the hot holes are injected into the gate oxide, Si–O and Si–H bonds can be damaged, leading to dangling bonds. As shown in Fig. 2b, the time exponent of the degradation at the early stress stage is high and then saturates with an increase in *t*_OSS_. Such a trend demonstrates that the defects generated by the OSS mostly originate from the broken Si–O bond.

In sensor applications, the LFN determines the SNR and LOD of the sensor, which requires a systematic investigation [18]. The LFN characteristics of the FET-type gas sensors are determined by the FET transducer, whose properties are strongly affected by the gate oxide-channel interface quality.^13^ When the carriers (electron) in the FET channel moves from source to drain, the carriers are trapped/detrapped to/from defects inside the gate oxide (SiO_2_ in this work). A time constant of trapping/detrapping process generates the Lorentzian noise with a specific corner frequency, and the distribution of traps with different time constants produces a power spectral density (PSD) of 1/*f* noise. This can be modeled using the carrier number fluctuation (CNF) model [13].

The CNF model is expressed as [24]

1 $$\frac{{S_{{{\text{ID}}}} }}{{I_{D}^{2} }} = \left( {\frac{{g_{m} }}{{I_{D} }}} \right)^{2} S_{{{\text{Vfb}}}}$$

with

2 $$S_{{{\text{Vfb}}}} = \frac{{q^{2} kTN_{T} \lambda }}{{WLC_{{{\text{ox}}}}^{2} f}}$$

where *g*_m_ is the transconductance, *S*_Vfb_ is the flat-band voltage noise spectral density, *q* is the electron charge, *k* is the Boltzmann constant, *T* is the temperature, *N*_T_ is the volume trap density, $\lambda$ is the tunneling attenuation coefficient in the gate oxide, *C*_ox_ is the gate oxide capacitance, *f* is the frequency. The *S*_Vfb_ is proportional to the *N*_T_ and inversely proportional to the dimension of the channel (*WL*). The reason why the *S*_Vfb_ is inversely proportional to *WL* is that the magnitude of noise is inversely proportional to the effective size of the noise source. The *S*_Vfb_ is reflected *S*_ID_ by the amplification of *g*_m_. In addition to CNF, the carrier mobility can be fluctuated by a trapped carrier in the gate oxide due to Coulomb interaction [25, 26]. This phenomenon is called correlated mobility fluctuation (CMF). The CNF model is modified by multiplying a CMF term ${(1\pm \alpha {\mu }_{\mathrm{eff}}{C}_{\mathrm{ox}}\frac{{I}_{D}}{{g}_{m}})}^{2}$ in Eq. (1). Note that *α* is the correlated mobility fluctuation parameter and *μ*_eff_ is the effective mobility.

**Fig. 3:**
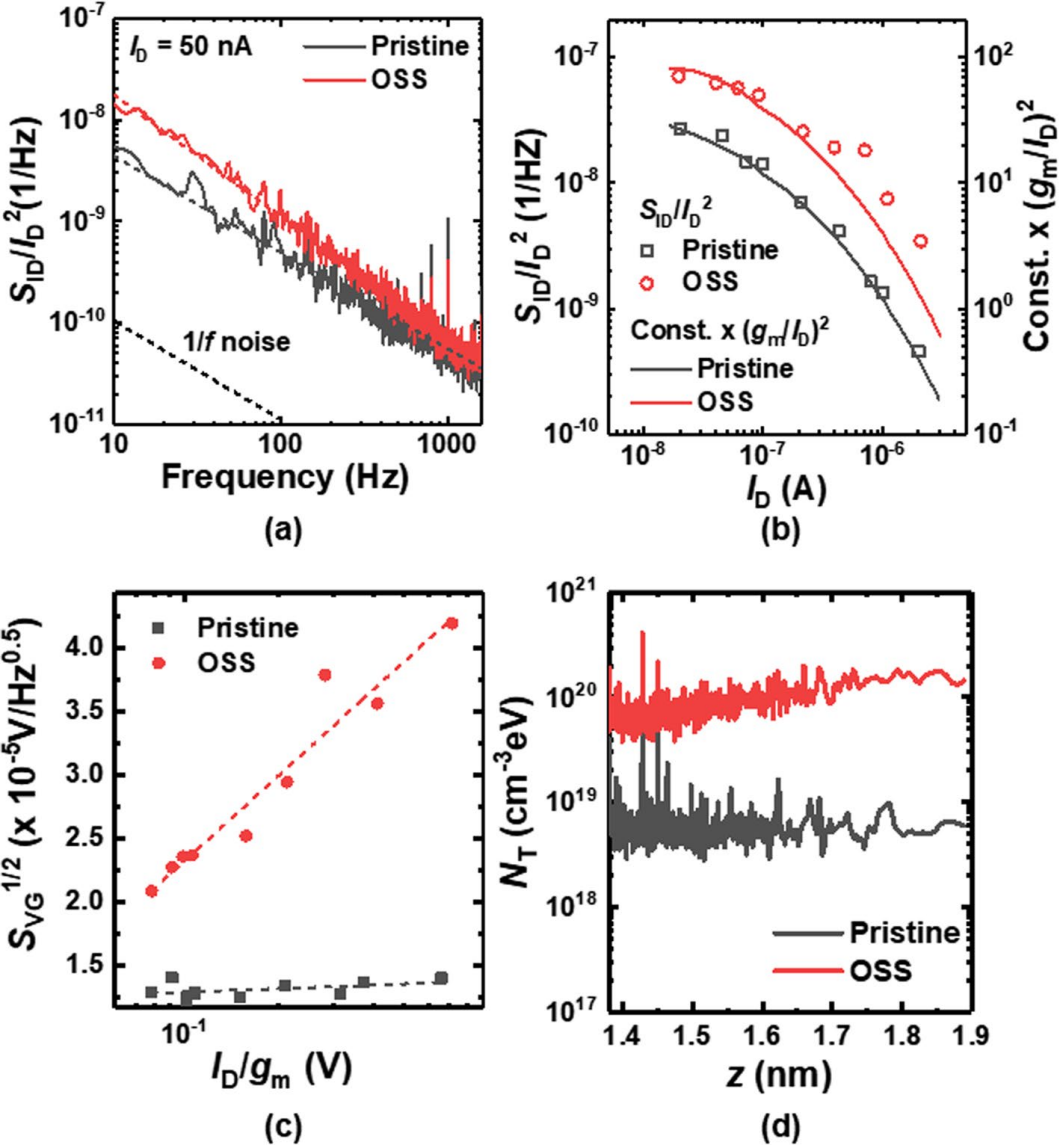
**a** *S*_ID_/*I*_D_^2^ of the sensor before (pristine) and after the OSS. **b** *S*_ID_/*I*_D_^2^ sampled at 1 Hz and constant $\times$ (*g*_m_/*I*_D_)^2^ versus *I*_D_ of the sensor before and after the OSS. **c** *S*_VG_^1/2^ versus *I*_D_/*g*_m_ of the sensor before and after the OSS. **d** *N*_T_ versus *z* of the sensor before and after the OSS

Figure 3a shows the normalized drain current power spectral density (*S*_ID_/*I*_D_^2^) of the before (pristine) and after the OSS. The 1/*f* noise is increased after the OSS. In order to find where such an increase stems from, the origin of 1/*f* noise is verified. As shown in Fig. 3b, the *S*_ID_/*I*_D_^2^ and (*g*_m_/*I*_D_)^2^ show similar behavior with respect to *I*_D_, demonstrating that the 1/*f* noise stems from the CNF [27, 28]. Figure 3c shows the input gate-voltage noise power spectral density (*S*_VG_) versus *I*_D_/*g*_m_. An increase in *S*_VG_ with *I*_D_/*g*_m_ after the OSS is due to the CMF. Figure 3d shows trap density (*N*_T_) along the vertical depth (*z*) of the gate oxide extracted in the sensor before and after the OSS. It is clearly observed that the *N*_T_ is increased after the OSS.

Now we evaluate the SNR of the sensors. In most studies that evaluate the LOD of gas sensors, LODs are determined by simply extrapolating the sensitivity to the gas concentration at which the response becomes either one or zero (depending on the definition of response employed). However, the LoD of sensors is also determined by the noise of the sensing signal. Therefore, the noise of the sensor should also be considered in evaluating LOD; thus, the signal-to-noise ratio (SNR) rather than the response is a more valid metric. Note that the noise of gas sensors is determined by LFN due to a long time response (from a few seconds to hundreds of seconds).In this study, the SNR is defined as

3 $$\mathrm{SNR}=\frac{\Delta I}{\delta I}=\frac{{I}_{g}-{I}_{a}}{\sqrt{{\int }_{f1}^{f2}{S}_{I}\left(f\right)df}}=\frac{{I}_{g}-{I}_{a}}{\sqrt{BW}\times \sqrt{{S}_{I}(f=1\,\text{Hz})}}$$

where Δ*I* is signal generated from the gas reaction; δ*Ι* is root-mean-square drain current noise amplitude; *S*_I_ (*f* = 1 Hz) is PSD of the current noise at a frequency of 1 Hz; and BW is a bandwidth-related term that depends on the largest (*f*_2_) and smallest (*f*_1_) frequencies sampled; *BW* = ln(*f*_2_/*f*_1_). Note that $\sqrt{BW}$ ranges from about 3.5 to 3.8 in typical gas sensing measurements. The LFN determines the δ*Ι* as the magnitude of the 1/*f* noise is in several orders larger than that of thermal noise in the measuring *BW* range. Therefore, an evaluation of SNR has to be based on the accurate measurement of the PSD of 1/*f* noise.

**Fig. 4:**
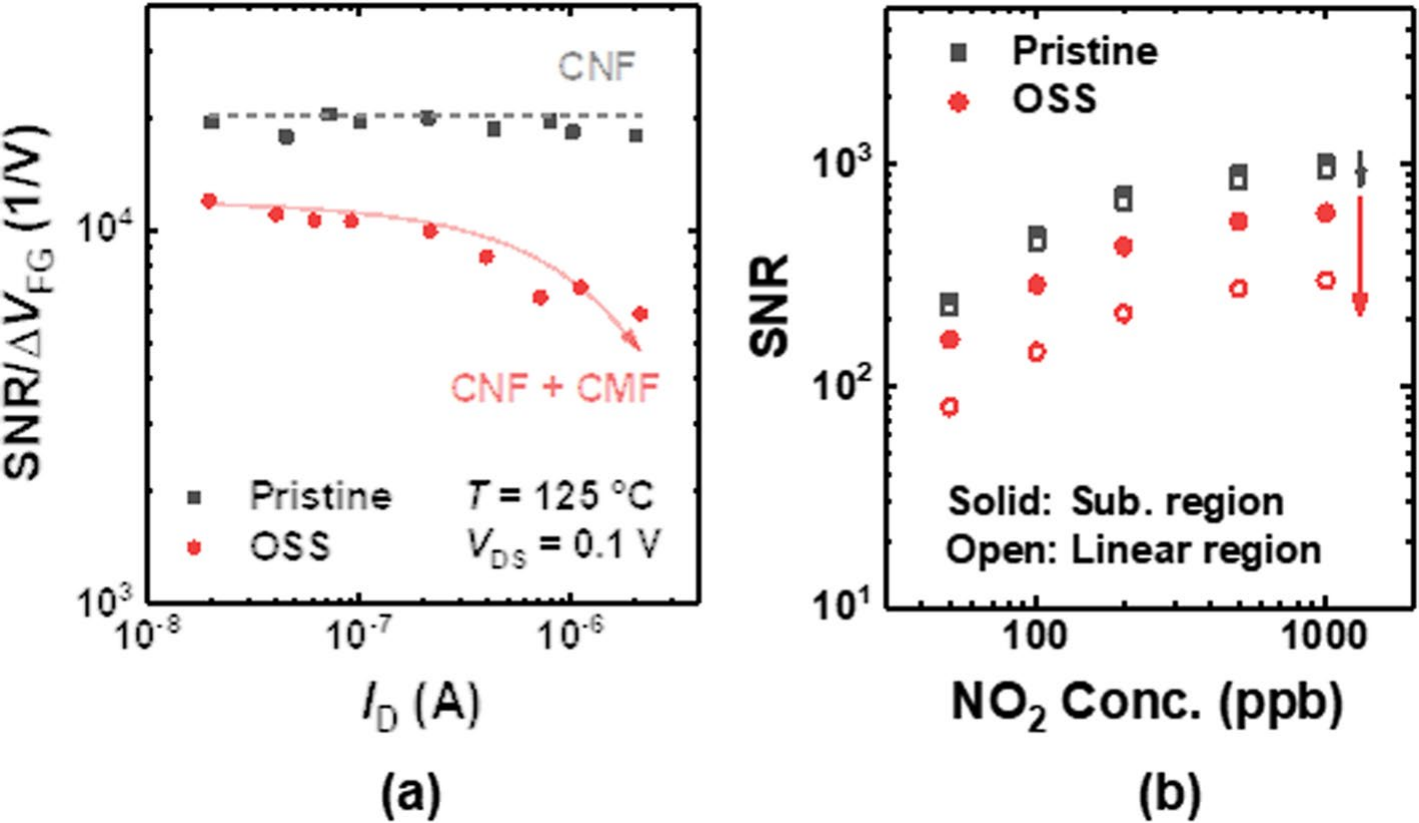
**a** SNR/Δ*V*_FG_ versus *I*_D_ of the sensor before and after the OSS. **b** SNR versus NO_2_ concentration of the sensor before and after the OSS at different operating regions

The SNR per unit change of FG voltage (Δ*V*_FG_) due to the NO_2_ gas reaction of the FET-type gas sensor is *g*_m_/$\sqrt{BW}\times \sqrt{{S}_{VG}}$ where *S*_VG_ is gate-voltage noise power spectral density [18, 29]. The Δ*V*_FG_ does not show a significant difference after the OSS because the Δ*V*_FG_ is determined by the sensing material, not by the FET transducer (Fig. 4b). Figure 4c shows the SNR/Δ*V*_FG_ versus *I*_D_ of the sensor before and after the OSS. It is revealed that the increased 1/*f* noise due to the OSS significantly decreases the SNR. A larger decrease in SNR in the linear region is due to the increased impact of CMF after the OSS. Figure 4d shows the SNR of the sensors versus NO_2_ gas concentration in different operating regions.

**Fig. 5:**
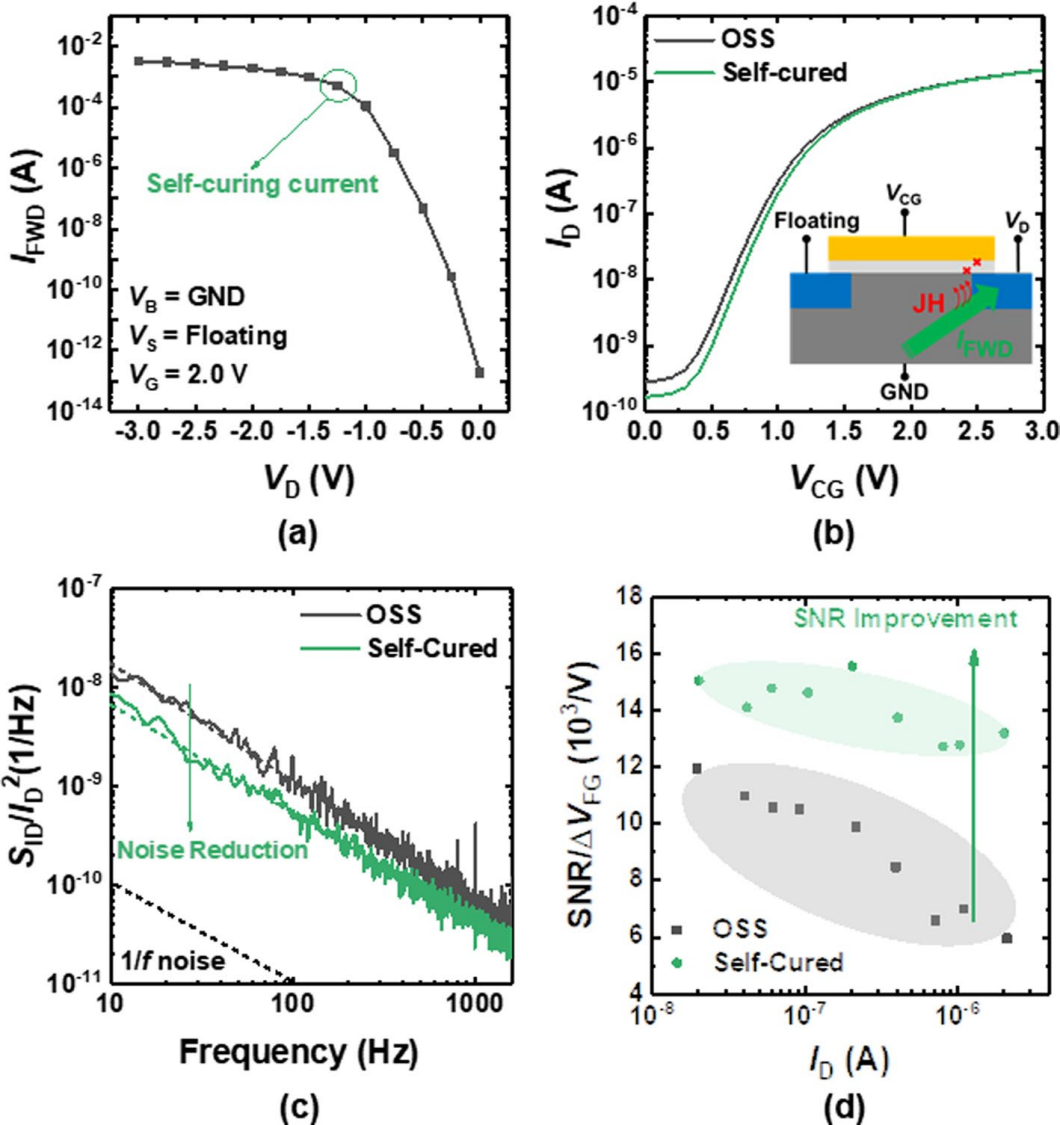
**a** *I*_FWD_ versus *V*_D_ of the sensor. **b** *I*_D_–*V*_CG_ of the off-state stressed and self-cured sensors. **c** *S*_ID_/*I*_D_^2^ of the off-state stressed and self-cured sensors. **d** SNR/Δ*V*_FG_ versus *I*_D_ of the off-state stressed and self-cured sensors

To improve the SNR degradation, the method to recover the damage originating from the OSS should be provided. Previous studies reported that the damage induced by the OSS can be cured by the thermal annealing [16]. However, the problem is that conventional thermal annealing using external equipment, including a furnace cannot be employed to repair OSS-caused damage because the heat treatment is difficult after being integrated on the system board. In contrast, the self-curing method employs Joule heat (JH) that is generated by the self-heating effect within the transistor itself [21–24]. Therefore, the self-curing method can be used selectively on the damaged device to recover the device even after packaging. In this study, the PN-junction forward current is used to generate JH in the transistor, thereby repairing the OSS-induced damage. When a large current is generated at the gate/drain overlap region by the PN-junction forward bias, the defects are cured by the JH from the forward junction current (*I*_FWD_). Figure 5a shows the *I*_FWD_ versus *V*_D_. As a very large *I*_FWD_ flows through the junction, the Joule heat (JH) is generated, which can be used to cure the damaged gate oxide. Figure 5b shows the *I*_D_–*V*_CG_ of the OSS-damaged and self-cured sensor. The inset schematically illustrates the mechanism of self-curing. Figure 5c shows the *S*_ID_/*I*_D_^2^ of the OSS-damaged and self-cured sensors. The 1/*f* noise is decreased by self-curing because the JH decreases the traps generated by the OSS. Figure 5d shows the SNR/Δ*V*_FG_ of the OSS-damaged and self-cured sensors. It is also revealed that the impact of CMF is decreased by self-curing.

Now we investigate the NO_2_ gas sensing properties of the sensors. When the sensor is exposed to NO_2_, the negative sheet chare (NO_2_^−^) is accumulated at the interface between the passivation layer and the sensing material. To compensate for the excessive NO_2_^−^ charge, the electrons are depleted at the channel of the FET transducer. Accordingly, the *I*_D_ of the sensor is decreased.

**Fig. 6:**
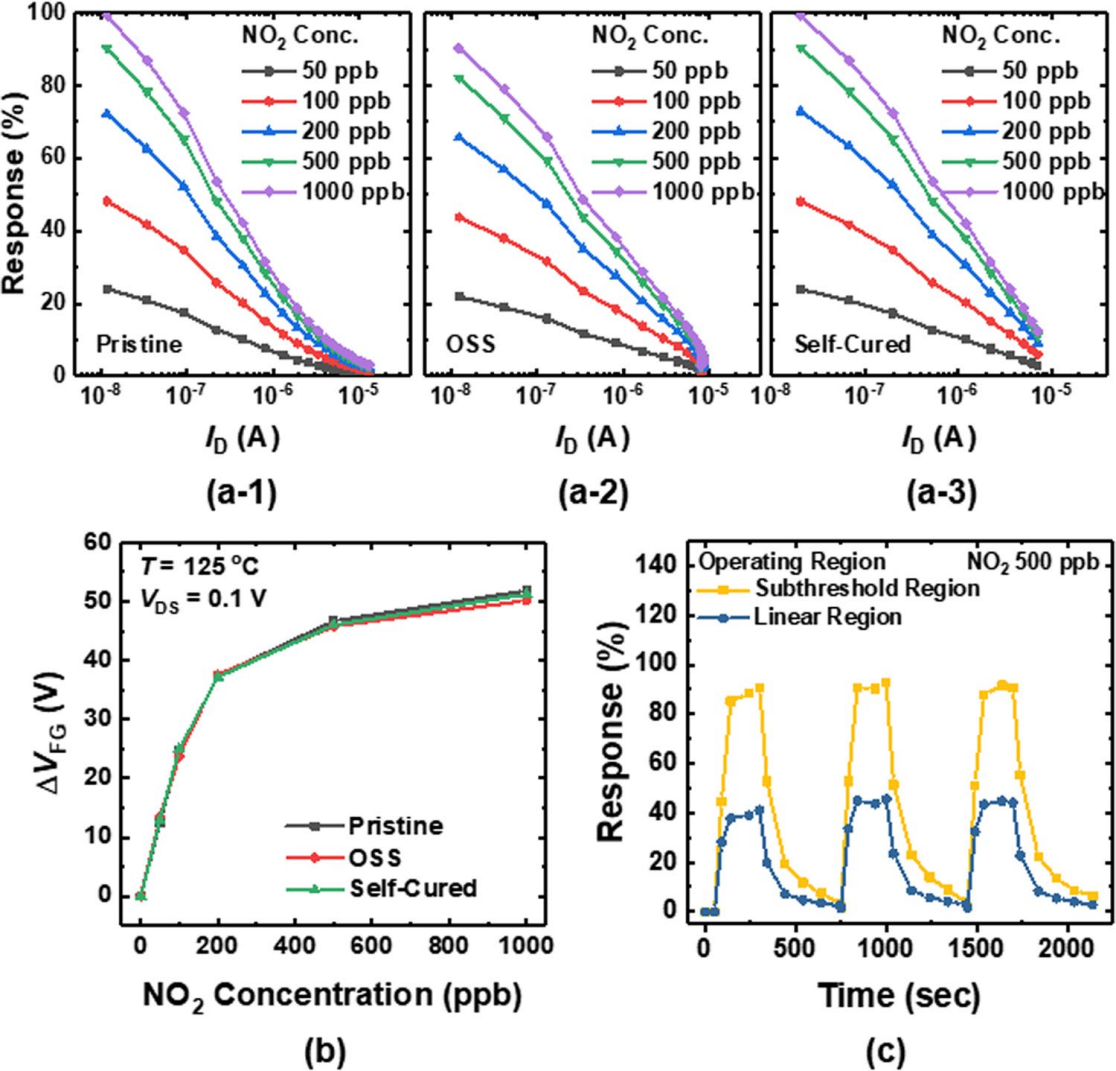
Response versus *I*_D_ of the (**a**-1) pristine, (**a**-2) OSS-damaged, and (**a**-3) self-cured sensors at various NO_2_ concentrations. **b** Δ*V*_FG_ versus NO_2_ concentration of the pristine, OSS-damaged, and self-cured sensors. **c** Transient response behaviors of the self-cured exposed to 500 ppb of NO_2_ gas. The transient behaviors are measured at the subthreshold and linear regions

Figures 6a-1, a-2, and a-3 correspondingly show the response versus *I*_D_ of the pristine, OSS-damaged, and self-cured sensors at different NO_2_ gas concentrations. The *V*_DS_ is fixed at 0.1 V, and *V*_CG_ is swept from 0.0 to 3.0 V. In this study, the response is defined as

4 $$\mathrm{Response }(\mathrm{\%})= \frac{\left|{I}_{\mathrm{D},\mathrm{air}}-{I}_{\mathrm{D},\mathrm{gas}}\right|}{{I}_{\mathrm{D},\mathrm{air}}} \times 100$$

where the *I*_D,air_ and *I*_D,gas_ are the *I*_D_ of the sensors at reference gas (dry air) and NO_2_ gas, respectively. Because the $\left|{I}_{\mathrm{D},\mathrm{air}}-{I}_{\mathrm{D},\mathrm{gas}}\right|$ is proportional to the *g*_m_ $\times$ Δ*V*_FG_ [18]_,_ the response can be expressed as

5 $$\mathrm{Response }(\mathrm{\%})= \frac{{g}_{m}}{{I}_{\mathrm{D},\mathrm{air}}} \times \Delta {V}_{\mathrm{FG}}\times 100$$

where Δ*V*_FG_ denotes the change in the FG voltage from the NO_2_ gas reaction. In all cases, the response exhibits its largest value at the subthreshold region owing to the largest transconductance efficiency (*g*_m_/*I*_D_). As shown in Fig. 6b, there is no change in Δ*V*_FG_ after the OSS. The result demonstrates that the OSS does not affect the properties of sensing materials (In_2_O_3_). However, the decrease in response is observed in Fig. 6a-2 after the OSS. This originates from the degradation in transconductance efficiency (*g*_m_/*I*_D_) due to the damaged gate oxide, as demonstrated in Fig. 3. From these results, it is revealed that not only the SNR but also the response of the sensors is degraded by the OSS. Figure 6c shows the transient response behaviors of the self-cured sensor exposed to 500 ppb of NO_2_ gas. The transient behaviors are measured at the subthreshold and linear regions. *I*_D_s of 80 and 800 nA are used to operate the sensor in the linear and subthreshold regions.

**Fig. 7:**
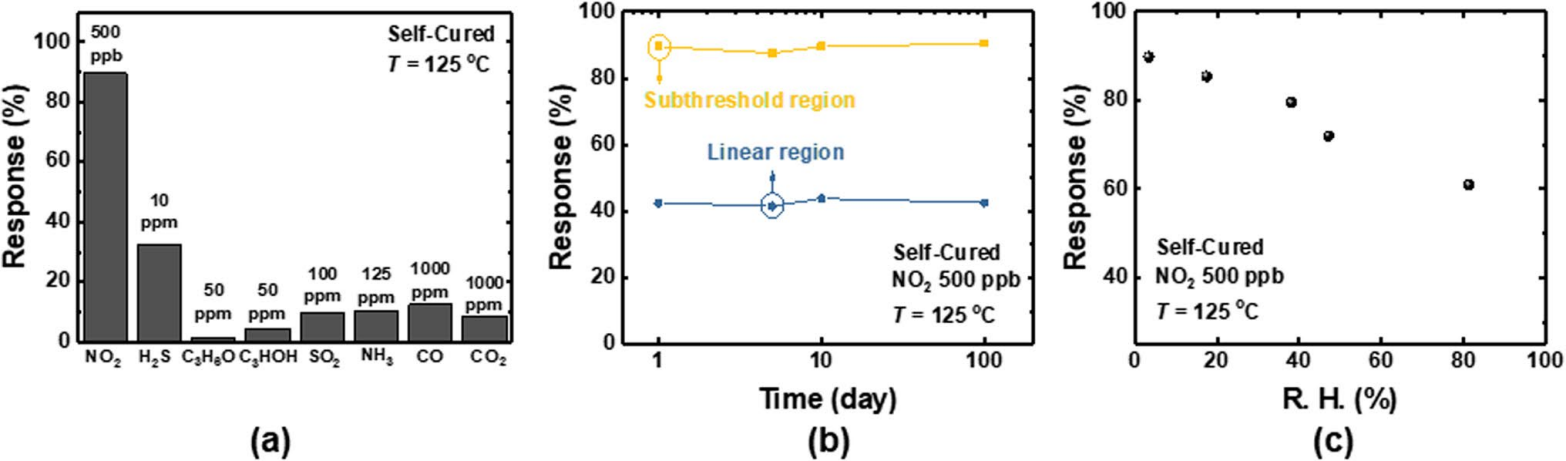
**a** Response of the self-cured sensor to different types of gases (NO_2_: 500 ppb, H_2_S: 10 ppm, C_3_H_6_O: 50 ppm, C_3_HOH: 50 ppm, SO_2_: 100 ppm, NH_3_: 125 ppm, CO: 1000 ppm, and CO_2_: 1000 ppm). **b** Long-term stability of the self-cured sensor. **c** Response of the self-cured sensor versus R. H.

Figure 7a shows the response of the sensor to different types of gases (NO_2_: 500 ppb, H_2_S: 10 ppm, C_3_H_6_O: 50 ppm, C_3_HOH: 50 ppm, SO_2_: 100 ppm, NH_3_: 125 ppm, CO: 1000 ppm, and CO_2_: 1000 ppm). The sensor shows the largest response to NO_2_ gas, exhibiting excellent selectivity. Figure 7b shows the long-term stability of the sensor. The response of the sensor is maintained for 100 days, demonstrating good long-term stability. Figure 7c shows the effects of humidity on the sensors. The humidifier produces humid air by passing dry air (3.4% RH) through it. The mass flow controller regulates the gas flow rates of the reference gas and the dry air going through the humidifier. The detailed measurement condition is explained in [30]. With an increase in relative humidity (R. H.) from 3.5 to 81.3%, the response decreases from 89.1 to 61%. It seems that the H_2_O molecules in humid air interfere with the interaction of gas molecules and In_2_O_3_ film.

## Conclusions

In this study, we investigated the impact of OSS on the NO_2_ gas sensing performance of the horizontal FG FET-type gas sensors. The LFN of the sensor is determined by the 1/*f* noise of the FET transducer, and its magnitude is significantly increased by damaged gate oxide due to the hot holes. Accordingly, the SNR of the sensor is significantly degraded by the OSS. Such degradation is repaired by the self-curing method based on forward current at the body–drain PN junction. The proposed self-curing method can be successfully adopted to recover the damage caused by the OSS. The results of this study pave the way to improve the reliability of the FET-type gas sensor by proposing an efficient self-curing method.

## Data Availability

The datasets used and/or analyzed during the current study are available from the corresponding author on reasonable request.

## References

[1] Su Y, Chen G, Chen C, Gong Q, Xie G, Yao M, Chen J (2021). Self-powered respiration monitoring enabled by a triboelectric nanogenerator. Adv Mater.

[2] Liu B, Libanor A, Zhou Y, Xiao X, Xie G, Zhao X, Chen J (2022). Simultaneous biomechanical and biochemical monitoring for self-powered breath analysis. ACS Appl Mater Interfaces.

[3] Chen C, Jiang M, Luo X, Tai H, Jiang Y, Yang M, Su Y (2022). Ni-Co-P hollow nanobricks enabled humidity sensor for respiratory analysis and human-machine interfacing. Sens Actuators B Chem.

[4] Fan H, Li H, Han J, McKeever N, Yu J, Katz HE (2019). A humid-air-operable, NO_2_-responsive polymer transistor series circuit with improved signal-to-drift ratio based on polymer semiconductor oxidation. ACS Sens.

[5] Yuan Z, Bariya M, Fahad HM, Wu J, Han R, Gupta N, Javey A (2020). Trace-level, multi-gas detection for food quality assessment based on decorated silicon transistor arrays. Adv Mater.

[6] Hong S, Wu M, Hong Y, Jeong Y, Jung G, Lee JH (2021). FET-type gas sensors: a review. Sens Actuators B Chem.

[7] Shin W, Yim J, Bae JH, Lee JK, Hong S, Kim J, Lee JH (2022). Synergistic improvement of sensing performance in ferroelectric transistor gas sensors using remnant polarization. Mater Horiz.

[8] Dey A (2018). Semiconductor metal oxide gas sensors: a review. Mater Sci Eng B.

[9] Han HJ, Lee GR, Xie Y, Jang H, Hynek DJ, Cho EN, Cha JJ (2021). Unconventional grain growth suppression in oxygen-rich metal oxide nanoribbons. Sci Adv.

[10] Postica V, Lupan O, Gapeeva A, Hansen L, Khaledialidusti R, Mishra AK, Hansen S (2021). Improved long-term stability and reduced humidity effect in gas sensing: SiO_2_ ultra-thin layered ZnO columnar films. Adv Mater Technol.

[11] Das S, Mojumder S, Saha D, Mrinal P (2022). Influence of major parameters on the sensing mechanism of semiconductor metal oxide based chemiresistive gas sensors: a review focused on personalized healthcare. Sens Actuators B Chem.

[12] Park J, Jung G, Hong S, Jeong Y, Lee JH (2022). Analysis of Cr/Au contact reliability in embedded poly-Si micro-heater for FET-type gas sensor. Sens Actuators B Chem.

[13] Shin W, Jung G, Hong S, Jeong Y, Park J, Jang D, Lee JH (2020). Low frequency noise characteristics of resistor-and Si MOSFET-type gas sensors fabricated on the same Si wafer with In_2_O_3_ sensing layer. Sens Actuators B Chem.

[14] Shin W, Kwon D, Ryu M, Kwon J, Hong S, Jeong Y, Lee JH (2021). Effects of IGZO film thickness on H2S gas sensing performance: Response, excessive recovery, low-frequency noise, and signal-to-noise ratio. Sens Actuators B Chem.

[15] Varghese D, Reddy V, Krishnan S, Alam MA (2014). OFF-state degradation and correlated gate dielectric breakdown in high voltage drain extended transistors: a review. Microelectron Reliab.

[16] Lee GB, Kim CK, Yoo MS, Hur J, Choi YK (2019). Effect of OFF-state stress on gate-induced drain leakage by interface traps in buried-gate FETs. IEEE Trans Electron Devices.

[17] Lee GB, Kim CK, Bang T, Yoo MS, Choi YK (2021). Lateral profiling of gate dielectric damage by off-state stress and positive-bias temperature instability. Microelectron Reliab.

[18] Shin W, Hong S, Jeong Y, Jung G, Park J, Kim D, Lee JH (2022). Effects of channel length scaling on the signal-to-noise ratio in FET-type gas sensor with horizontal floating-gate. IEEE Electron Device Lett.

[19] Shin W, Jung G, Hong S, Jeong Y, Park J, Kim D, Lee JH (2022). Optimization of channel structure and bias condition for signal-to-noise ratio improvement in Si-based FET-type gas sensor with horizontal floating-gate. Sens Actuators B Chem.

[20] Shin W, Hong S, Jung G, Jeong Y, Park J, Kim D, Lee JH (2021). Improved signal-to-noise-ratio of FET-type gas sensors using body bias control and embedded micro-heater. Sens Actuators B Chem.

[21] Lee GB, Jung JW, Kim CK, Bang T, Yoo MS, Choi YK (2021). Improved self-curing effect in a MOSFET with gate biasing. IEEE Electron Device Lett.

[22] Park JY, Moon DI, Lee GB, Choi YK (2020). Curing of aged gate dielectric by the self-heating effect in MOSFETs. IEEE Trans Electron Devices.

[23] Shin W, Koo RH, Hong S, Kwon D, Hwang J, Park BG, Lee JH (2022). Highly efficient self-curing method in MOSFET using parasitic bipolar junction transistor. IEEE Electron Device Lett.

[24] Kim HJ, Lee GB, Han JK, Han SJ, Kim DJ, Yu JM, Choi YK (2022). Curing of 1-transistor-DRAM by joule heat from punch-through current. IEEE Electron Device Lett.

[25] Ghibaudo G, Roux O, Nguyen-Duc C, Balestra F, Brini J (1991). Improved analysis of low frequency noise in field-effect MOS transistors. Phys Status Solidi (a).

[26] Rehman A, Notario JAD, Sanchez JS, Meziani YM, Cywiński G, Knap W, Rumyantsev S (2022). Nature of the 1/*f* noise in graphene—direct evidence for the mobility fluctuation mechanism. Nanoscale.

[27] Shin W, Kwon D, Bae JH, Lim S, Park BG, Lee JH (2021). Impacts of program/erase cycling on the low-frequency noise characteristics of reconfigurable gated Schottky diodes. IEEE Electron Device Lett.

[28] Shin W, Jung G, Hong S, Jeong Y, Park J, Kim D, Lee JH (2020). Proposition of deposition and bias conditions for optimal signal-to-noise-ratio in resistor-and FET-type gas sensors. Nanoscale.

[29] Hong S, Jeong Y, Jung G, Park J, Kim D, Lee JH (2021). Effect of charge storage engineering on the NO_2_ gas sensing properties of a WO_3_ FET-type gas sensor with a horizontal floating-gate. Nanoscale.

[30] Jeong Y, Hong S, Jung G, Shin W, Park J, Kim D, Lee JH (2021). Highly stable Si MOSFET-type humidity sensor with ink-jet printed graphene quantum dots sensing layer. Sens Actuators B Chem.

